# Is There an Economical Running Technique? A Review of Modifiable Biomechanical Factors Affecting Running Economy

**DOI:** 10.1007/s40279-016-0474-4

**Published:** 2016-01-27

**Authors:** Isabel S. Moore

**Affiliations:** Cardiff School of Sport, Cardiff Metropolitan University, Cardiff, CF23 6XD Wales, UK

## Abstract

Running economy (RE) has a strong relationship with running performance, and modifiable running biomechanics are a determining factor of RE. The purposes of this review were to (1) examine the intrinsic and extrinsic modifiable biomechanical factors affecting RE; (2) assess training-induced changes in RE and running biomechanics; (3) evaluate whether an economical running technique can be recommended and; (4) discuss potential areas for future research. Based on current evidence, the intrinsic factors that appeared beneficial for RE were using a preferred stride length range, which allows for stride length deviations up to 3 % shorter than preferred stride length; lower vertical oscillation; greater leg stiffness; low lower limb moment of inertia; less leg extension at toe-off; larger stride angles; alignment of the ground reaction force and leg axis during propulsion; maintaining arm swing; low thigh antagonist–agonist muscular coactivation; and low activation of lower limb muscles during propulsion. Extrinsic factors associated with a better RE were a firm, compliant shoe–surface interaction and being barefoot or wearing lightweight shoes. Several other modifiable biomechanical factors presented inconsistent relationships with RE. Running biomechanics during ground contact appeared to play an important role, specifically those during propulsion. Therefore, this phase has the strongest direct links with RE. Recurring methodological problems exist within the literature, such as cross-comparisons, assessing variables in isolation, and acute to short-term interventions. Therefore, recommending a general economical running technique should be approached with caution. Future work should focus on interdisciplinary longitudinal investigations combining RE, kinematics, kinetics, and neuromuscular and anatomical aspects, as well as applying a synergistic approach to understanding the role of kinetics.

## Key Points

**Table:** 

Running biomechanics during ground contact, particularly those related to propulsion, such as less leg extension at toe-off, larger stride angles, alignment of the ground reaction force and leg axis, and low activation of the lower limb muscles, appear to have the strongest direct links with running economy.
Inconsistent findings and limited understanding still exist for several spatiotemporal, kinematic, kinetic, and neuromuscular factors and how they relate to running economy.

## Introduction

For competitive runners, decreasing the time needed to complete a race distance is crucial. Consequently, there is a need to understand the determinants of running performance. Several physiological determinants have been identified, which include a high maximal oxygen uptake ($\dot{V}{\text{O}}_{{ 2 {\text{max}}}}$) [1, 2], lactate threshold [3, 4], and running economy (RE) [5, 6].

In a heterogeneous group of runners, $\dot{V}{\text{O}}_{{ 2 {\text{max}}}}$ is strongly related to running performance [7]. However, in a group of runners with a similar $\dot{V}{\text{O}}_{{ 2 {\text{max}}}}$, $\dot{V}{\text{O}}_{{ 2 {\text{max}}}}$ cannot be used to discern between those who out-perform others [6]. A measure that can distinguish between good and poor running performers is the rate of oxygen consumed at a given submaximal running velocity, termed RE [5, 8, 9], with lower oxygen consumption ($\dot{V}{\text{O}}_{2}$) indicating better RE during steady-state running. For a group of runners with a similar $\dot{V}{\text{O}}_{{ 2 {\text{max}}}}$, RE can differ by as much as 30 % and is a better predictor of running performance than $\dot{V}{\text{O}}_{{ 2 {\text{max}}}}$ [6, 8, 10]. Several researchers have reported strong associations between RE and running performance [5, 7, 11, 12]. Additionally, RE differs substantially between elite, trained (recreational), and untrained runners and also between males and females [13–17]. Saunders et al. [18] proposed the following determinants of RE: training, environment, physiology, anthropometry, and running biomechanics.

Studies utilizing interventions show RE can be improved [19], meaning it is a ‘trainable’ parameter [20]. Improvements in RE have ranged from 2 to 8 % using various short-term training modes, such as plyometric [21–23], strength and resistance [24–27], whole-body vibration [28], interval [29–31], altitude [32, 33], and endurance running [34, 35]. In comparison, long-term physiological training can improve RE by 15 % [12]. Jones [12] reported that such an improvement over 9 years was probably a crucial factor in the elite marathon runner’s continued improvement in running performance. For intervention studies concerned with improving RE, the initial fitness level of participants is particularly important [18], with a high initial fitness level perhaps explaining why not all interventions have successfully improved RE [36–39]. Nevertheless, the trainability of RE suggests certain factors affecting RE can be modified. One such factor that can influence RE is an individual’s running biomechanics.

Understanding what constitutes an economical running technique has been the focus of much research. Specific factors include spatiotemporal factors [40, 41], lower limb kinematics [34, 42], kinetics [9, 43, 44], neuromuscular factors [45–48], the shoe–surface interaction [49–54], and trunk and upper limb biomechanics [55–57]. Synthesizing the literature within this field of research has received limited attention, with some still drawing upon descriptors provided up to 20 years ago [18, 58]. Much research has been conducted since, in an attempt to answer the question: is there an economical running technique? Therefore, the purposes of this review are to (1) examine the intrinsic and extrinsic modifiable biomechanical factors affecting RE; (2) assess training-induced changes in RE and running biomechanics; (3) evaluate whether an economical running technique can be recommended; and (4) discuss potential areas for future research directions.

## Modifiable Biomechanical Factors Affecting Running Economy

Several modifiable biomechanical factors may affect RE. Each factor can be considered either intrinsic (internal) or extrinsic (external). Intrinsic factors refer to an individual’s running biomechanics. These factors can be further categorised as spatiotemporal (parameters relating to changes in and/or phases of the gait cycle, such as ground contact time and stride length); kinematics (the movement patterns, such as lower limb joint angles); kinetics (the forces that cause motion, such as ground reaction force [GRF]); and neuromuscular (the nerves and muscles, such as the activation and coactivation of muscles). The extrinsic factors covered in this review relate to the shoe–surface interaction and focus on footwear, orthotics, and running surface. Evidence for how each factor affects RE is reviewed and discussed.

## Spatiotemporal Factors

Stride frequency and stride length are mutually dependent and define running speed. If running speed is kept constant, increasing either stride frequency or stride length will result in a decrease of the other. Runners appear to naturally choose a stride frequency or stride length that is economically optimal, or at least very near to being economically optimal. This innate, subconscious fine-tuning of running biomechanics is referred to as self-optimization [34, 42]. Studies supporting this self-optimizing theory generally use acute manipulations of stride frequency or stride length and mathematical curve-fitting procedures to derive the most economical stride frequency and length [40, 59–61].

**Fig. 1:**
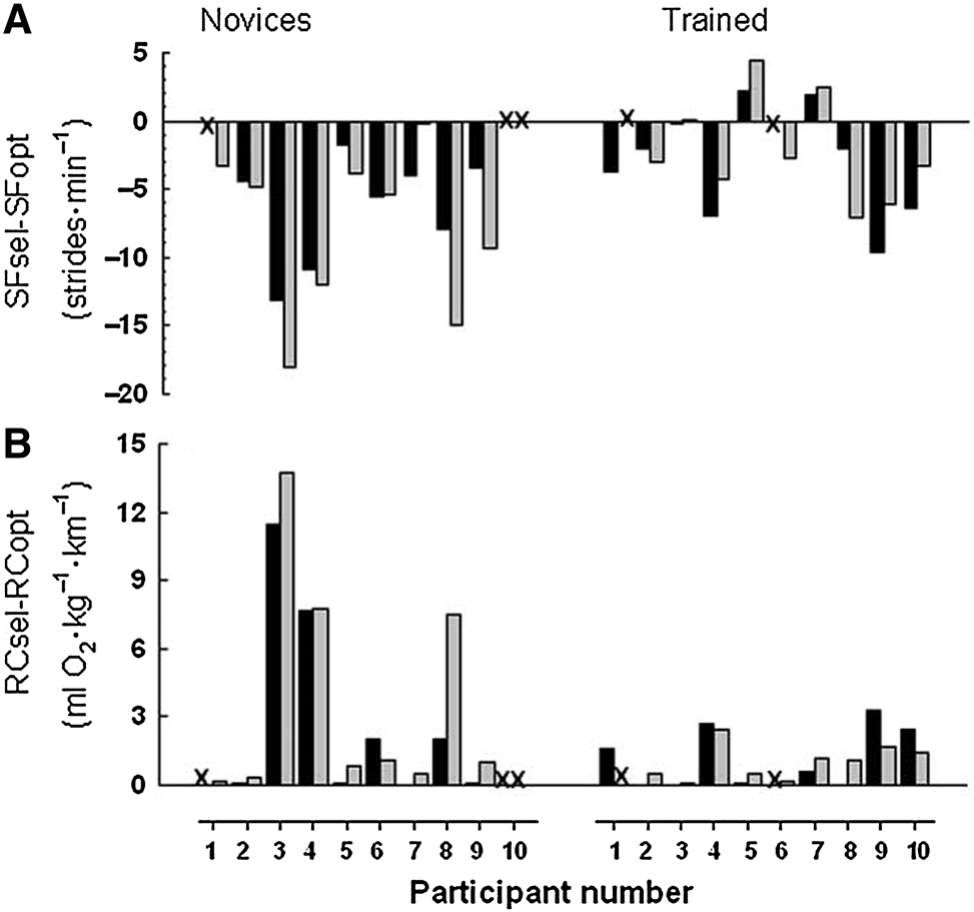
Individual differences (selected-optimal) in stride frequency (**a**) and running cost (**b**) for novice (*left*) and trained runners (*right*) on day 1 (*black bars*) and day 2 (*grey bars*). 2 test days were used to assess the reliability of measures and were separated by at least 48 h. *RCopt* running cost of optimal stride frequency, *RCsel* running cost of self-selected stride frequency, *SFopt* optimal stride frequency based on minimal running cost, *SFsel* self-selected stride frequency. *X* denotes that optimal stride frequency and, consequently, optimal running cost could not be established in these five trials. Reproduced from de Ruiter et al. [59] by permission of Taylor & Francis Ltd, http://www.tandfonline.com

Interestingly, a trained runner’s mathematical optimal stride frequency or stride length is, on average, 3 % faster or 3 % shorter than their preferred frequency or length [40, 59, 61]. Acute and short-term manipulations whereby stride length has been shortened by 3 % show RE to be unaffected [50, 62], whereas stride length deviations greater than 6 % are detrimental to RE [59]. Collectively, these results suggest there is an optimal stride length ‘range’ that trained runners can acutely adopt without compromising their RE. This range appears to be the preferred stride length minus 3 % to the preferred stride length. Importantly, even in a fatigued state, trained runners reduce their stride frequency compared with a non-fatigued state and produce a preferred stride frequency that is similar to their optimal stride frequency achieved in a fatigued state [60]. These results imply that trained runners can dynamically self-optimize their running biomechanics in response to their physiological state. For novice runners, the difference between preferred and mathematically optimal stride frequencies is greater than for trained runners (8 vs. 3 %) [59] (Fig. 1). Therefore, generalizing the principle of an optimal stride length range to all runners should be done with caution, as self-optimization appears to be a physiological adaptation resulting from greater running experience.

Similar to stride frequency and stride length, vertical oscillation can be altered. Acute interventions have shown that increasing vertical oscillation leads to increases in $\dot{V}{\text{O}}_{2}$ [41, 63]. Additionally, vertical oscillation increases when running to exhaustion. However, vertical oscillation changes are minimal and increases in $\dot{V}{\text{O}}_{2}$ are large [64, 65], meaning several other physiological and biomechanical factors contribute to increases in $\dot{V}{\text{O}}_{2}$ during fatigue [66, 67]. Furthermore, decreases in vertical oscillation have been shown when individuals run barefoot and their RE improves [50], probably due to a smaller vertical displacement during stance [52]. Yet, it must be noted that shoe mass and other biomechanical changes associated with barefoot running also influence such RE improvements (see Sect. 3.4). Another study has shown that decreasing vertical oscillation can slightly improve RE, but only if the absolute height of the body’s center of mass (CoM) is not changed [68]. Collectively, these results imply that reducing the magnitude of vertical displacement should be encouraged. It is possible that reducing vertical displacement improves RE by reducing the metabolic cost associated with supporting body weight, as a smaller vertical impulse would be produced [69]. Additionally, it could make a runner more mechanically efficient, as a low displacement of the body’s CoM produces a low mechanical energy cost, since the body is performing less work against gravity [70].

Notwithstanding these encouraging results, findings show that female runners have a lower vertical oscillation than their male counterparts, but findings are conflicting regarding whether females are more or less economical than males [13, 16, 71]. Eriksson et al. [72] demonstrated that vertical oscillation could be successfully lowered using visual and auditory feedback, and that runners found it more natural to change vertical oscillation than step frequency. However, to date, only one study has assessed the effect of specifically decreasing a runner’s vertical oscillation. This means research has not tried to manipulate vertical oscillation, in a similar manner to stride frequency and stride length, to determine whether runners have an optimal magnitude of vertical oscillation or whether runners would simply benefit from lowering their vertical oscillation to improve RE.

The time the foot spends in contact with the ground has equivocal results regarding its association with RE. Several studies have failed to find any relationship between ground contact time and RE [9, 42, 73, 74], whilst some have observed a better RE to be associated with longer contact times [75, 76] and others have found the opposite to be true [11, 77]. It is suggested that short ground-contact times incur a high metabolic cost because faster force production is required, meaning metabolically expensive fast twitch muscle fibers are recruited [78, 79]. Conversely, long ground-contact times may incur a high metabolic cost because force is produced slowly, meaning longer braking phases when runners undergo deceleration [77]. Whilst both arguments appear plausible, it has been argued that being able to reduce the amount of speed lost during ground contact is the most important aspect rather than the time in contact with it [77, 80–82]. Combining this with evidence that individuals can produce shorter ground-contact times, but similar deceleration times and RE when forefoot striking compared with rearfoot striking [83], suggests that the time spent decelerating may influence RE.

Another factor that may affect the body’s deceleration is how far ahead of the body the foot strikes the ground. Evidence from step rate manipulation investigations and global gait re-training studies instructing runners to adopt a Pose running method, suggest that significantly decreasing the horizontal distance between the body’s CoM and foot at initial ground contact reduces peak braking and propulsive forces [84, 85] and braking impulses (less speed lost) applied by the runner [86, 87]. Yet, both performance and RE were unaffected during the gait re-training [85], potentially because too many running biomechanics were modified at once. Others have suggested that a runner’s optimal stride frequency is a trade-off between the metabolic cost associated with braking impulses and those associated with swinging the leg [87]. Further work into this braking strategy is required to understand the implications for RE.

Increasing the absolute time spent in the swing phase has been associated with better RE by several researchers [11, 42, 43]. However, others have failed to find any relationship between the two [43, 71]. Findings from Barnes et al. [43] suggest that sex also affects this relationship; however, this has not been corroborated by others [11, 71]. It is conceivable that a longer absolute swing time means runners spend a smaller proportion of the gait cycle in contact with the ground, which is believed to be the metabolically expensive phase of the cycle. It is important to note that the swing and ground contact times will impact the stride frequency and stride length of a runner, and it is perhaps the relationship between all these aspects that should be considered.

### Lower Limb Kinematic Factors

Various kinematic parameters have been identified as being associated with better RE in cross-comparison studies; greater plantarflexion velocity [75], greater horizontal heel velocity at initial contact [75], greater maximal thigh extension angle with the vertical [75], greater knee flexion during stance [42], reduced knee range of motion during stance [88], reduced peak hip flexion during braking [88], slower knee flexion velocity during swing [42, 71], greater dorsiflexion and faster dorsiflexion velocity during stance [71], slower dorsiflexion velocity during stance [88], and greater shank angle at initial contact [42]. Intra-individual comparisons have identified later occurrence of peak dorsiflexion, slower eversion velocity at initial contact, and less knee flexion at push-off as being associated with improved RE [34].

**Fig. 2:**
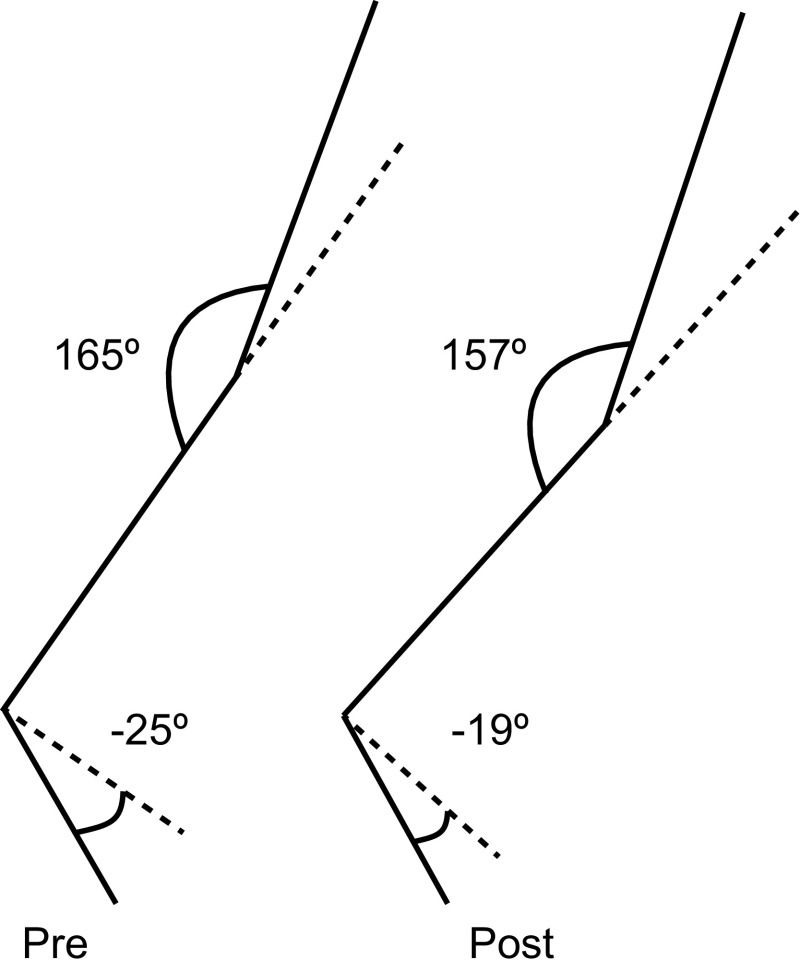
Differences in knee angle (*top*) and ankle angle (*bottom*) at toe-off between pre and post measurements. Pre refers to baseline running biomechanics and post refers to running biomechanics after 10 weeks of running whereby beginner runners improved their running economy and altered their running technique. Reproduced from Moore et al. [34], with permission

One of the few kinematic variables to have strong support from both cross- and intra-individual comparisons as being beneficial for RE is a less extended leg at toe-off [34, 42, 50, 71, 75, 89]. Evidence has shown that this can be achieved through less plantarflexion and/or less knee extension as the runner pushes off the ground (Fig. 2). Hip extension is also likely to contribute, but studies have typically focused on the knee and ankle angles. Less leg extension could produce greater propulsive force, as identified by Moore et al. [34], by potentially allowing the leg extensor muscles to operate at a more favorable position on the force–length curve and higher gear ratios (GRF moment arm to muscle–tendon moment arm) being obtained. Both strategies could maximize force production [90, 91]. Additionally, less leg extension would reduce the amount of flexion needed during swing by already being partially flexed and potentially reduce the leg’s moment of inertia, lowering the energy required to flex the leg during the swing phase. Previous research has shown that reduced leg moment of inertia lowers the leg’s mechanical demand during the swing phase, as well as the metabolic demand, of walking [92]. Therefore, it is conceivable that a similar relationship exists when running, but this needs investigating.

Another kinematic during the push-off phase that has been associated with better RE is stride angle, which is defined as the angle of the parable tangent of the CoM at toe-off [11, 93, 94]. Larger stride angles appear to be beneficial for lowering $\dot{V}{\text{O}}_{2}$ and can be achieved by either increasing swing time or decreasing stride length. However, the system (Optojump Next) used by each study [11, 93, 94] only tracks the foot during ground contact and not the CoM. Therefore, only inferences can be made regarding the trajectory angle of the CoM and other possible kinematic changes. Future work focusing on the push-off phase should assess CoM trajectory in relation to kinematics and kinetics, as increasing swing time would also increase the vertical displacement of the CoM based on previous calculations [11, 93, 94] and observations [95]. Crucially, research suggests that increases to these spatiotemporal parameters appear to have contradictory relationships with RE [11, 41–43, 63].

Foot strike patterns have been implicated as a modifiable factor affecting RE [96], with some researchers arguing that the most economical strike pattern is forefoot striking, even when RE is not assessed [97–99]. However, empirical evidence refutes this claim. Findings shows no difference in RE between rearfoot and forefoot striking at slow (≤3 m·s^−1^) [51, 83, 100, 101], medium (3.1–3.9 m·s^−1^) [83, 100, 101], and fast speeds (≥4.0 m·s^−1^) [83, 100] or rearfoot and midfoot striking at medium speeds [76]. However, others have shown rearfoot striking to be more economical than midfoot striking at slow running speeds [102]. Interestingly, habitual forefoot strikers can change to a rearfoot strike without detrimental consequences to RE, while an imposed forefoot strike in habitual rearfoot strikers produces worse RE at slow and medium speeds [100]. Based on the current literature, foot strike appears to have a negligible effect upon RE, with only habitual rearfoot strikers likely to experience a worsening of RE by switching foot strike patterns.

### Kinetic Factors

Early research reported that RE was proportional to the vertical component of GRF (e.g., force required to support body weight) and was termed the ‘cost of generating force’ hypothesis [79, 103, 104]. However, later investigations have used a task-by-task approach to partition RE into individual biomechanical tasks [105]. Such work has demonstrated that braking (decelerating the body) and propulsive (accelerating the body) forces also incur metabolic costs [105]. Typically, the three components of GRF (anterior-posterior, medial–lateral, and vertical) have been independently assessed, with evidence suggesting lower vertical impact force [42], lower peak medial–lateral force [42, 75], lower anterior–posterior braking force [73], and higher anterior–posterior propulsive force [34] are economical. However, numerous studies have also failed to identify similar associations between RE and individual GRF components [26, 73, 74].

**Fig. 3:**
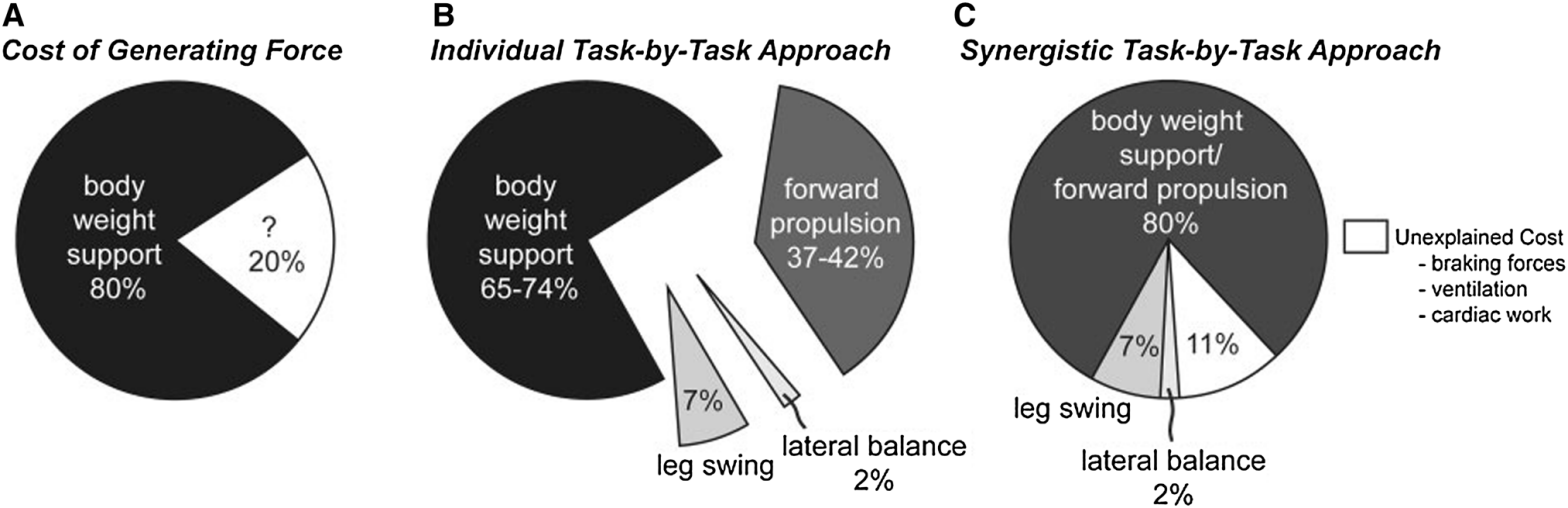
The **a** cost of generating force, **b** individual task-by-task, and **c** synergistic task-by-task approach partition the net metabolic cost of human running into its biomechanical constituents. The cost of generating force approach and the individual task-by-task approach both illustrate that body weight support is the primary determinant of the net metabolic cost of human running. In the individual task-by-task approach, forward propulsion represents the second largest determinant. The individual task-by-task approach leads to an overestimation, while the synergistic task-by-task approach suggests that the synergistic tasks of body weight support and forward propulsion are the primary determinants of the net metabolic cost of human running. Note that leg swing and lateral balance exact a relatively small net metabolic cost. If we sum all the biomechanical tasks, the synergistic task-by-task approach accounts for 89 % of the net metabolic cost of human running, leaving 11 % of unexplained metabolic cost, and the cost of generating force accounts for 80 %, leaving 20 % of unexplained metabolic cost. Reproduced from Arellano and Kram [106], with permission from Oxford University Press

To understand the metabolic costs incurred during running Arellano and Kram [106] advocate using a synergistic approach, rather than the ‘cost of generating force’ hypothesis or task-by-task approach. Using this approach, the vertical force (supporting body weight) and forward propulsive force (accelerating the body) incur the greatest metabolic cost (Fig. 3). However, very few biomechanical studies have utilized such an approach. Storen et al. [74] demonstrated that it could be usefully applied as they found significant relationships between the summation of peak vertical and anterior–posterior forces and 3-km performance (*r* = −0.71) and RE (*r* = −0.66). Their findings show that lower forces were associated with a better running performance and RE. Additionally, Moore et al. [107] reported near perfect alignment of the angle of the resultant GRF vector (all three components) with the angle of the longitudinal leg axis vector during propulsion when novice runners improved their RE. This change in alignment was associated with a change in RE (*r*_s_ = 0.88), suggesting that minimizing the muscular effort of generating force during propulsion is beneficial to RE [107].

Associations have also been found between GRF impulses and RE, with lower braking [87], total, and net vertical impulses related to a better RE [9]. However, this finding is not consistent in the literature [77]. Through collectively considering the deceleration and acceleration (anterior–posterior) impulses, a runner’s change in momentum can be determined. One pilot study has utilized this technique, but reported similar changes in momentum pre and post a 10-week running program that improved RE [107]. The authors suggested that such a short-term training program might not have been long enough to induce modifications in momentum [107]. It is also conceivable that a synergistic approach should be applied to momentum and speed lost during braking.

The magnitude of the GRF during running has a linear relationship with the body’s vertical displacement [108], suggesting the leg acts like a spring during ground contact [44]. Therefore, use of the spring-mass model to describe the body’s bounce during the support phase of running has been widespread. The springs’ stiffness is the ratio of deformation (vertical displacement) to the force applied to it (vertical GRF) and therefore represents the stiffness of the whole body’s musculoskeletal system [109]. Leg stiffness represents the ratio of maximal vertical force to maximal vertical leg spring compression [110]. Greater leg stiffness has been associated with a better RE [44], whilst fatiguing runs to volitional exhaustion have led to reductions in leg stiffness [64, 65]. Furthermore, alterations to extrinsic factors, such as increasing surface compliance, can lead to decreases in leg stiffness, resulting in a worse RE [111]. Running in minimalist footwear can increase leg stiffness and improve RE compared with traditional and cushioned footwear [112, 113]. Interestingly, leg stiffness is predominately associated with ground-contact time rather than step frequency [114]. Thus, to try and increase leg stiffness, runners are advised to focus on shortening ground-contact time rather than increasing step frequency. Such an approach may be beneficial for RE improvements.

As leg stiffness represents the stiffness of the whole musculoskeletal system, several factors relating to stiffness are unmodifiable, such as muscle crossbridges and tendon stiffness. However, neuromuscular activation is a modifiable characteristic that can modulate stiffness.

### Neuromuscular Factors

The preactivation of muscles prior to ground contact, termed muscle tuning, is believed to increase muscle–tendon stiffness [77], potentially enhance muscular force generation via the stretch–shortening cycle (SSC) [115], and affect leg geometry at initial ground contact [116–118]. Nigg et al. [119] studied the effect of shoe midsole characteristics on RE and preactivation, and, whilst no overall shoe-dependent changes were found in either variable, systematic individual changes in vastus medialis preactivation were evident. Runners who produced higher vastus medialis preactivation independent of shoe condition also had a higher $\dot{V}{\text{O}}_{2}$ [119]. However, given the small changes in RE (<2 %) the differences may be due to test–retest measurement error and are unlikely to represent a meaningful change in RE [120].

Greater muscular activity of the lower limbs has been reported as a potential mechanism behind increasing $\dot{V}{\text{O}}_{2}$ and thus is seen as detrimental to RE [73]. The intuitive link between muscle activity and RE stems from muscles needing to utilize oxygen to activate, and thereby, control movement patterns and stabilize joints. Therefore, greater muscle activation, as typically measured using surface electromyography (EMG), is thought to require a higher $\dot{V}{\text{O}}_{2}$ and lead to a worsening of RE. In line with this, findings have shown a higher activation of the gastrocnemius during propulsion and of the biceps femoris during braking and propulsion to be associated with higher $\dot{V}{\text{O}}_{2}$ [73]. Additionally, Abe et al. [45] found an increase in $\dot{V}{\text{O}}_{2}$ during a prolonged run was associated with a decrease in the ratio of eccentric–concentric vastus lateralis activity. This change in eccentric–concentric ratio was due to an increase in activity during propulsion (concentric phase). Collectively, these findings suggest that needing to utilize greater muscle activation to propel the runner forwards, possibly due to a reduced efficiency of the SSC, is detrimental to RE.

Bourdin et al. [121] support this notion, as they found lower eccentric–concentric ratios of vastus lateralis activity were associated with a higher energetic cost of running. Importantly, however, this relationship was more prominent when inter-individual differences were being assessed and was weaker when intra-individual differences were considered. Sinclair et al. [88] also found a higher activity of the vastus medialis to be related to a worse RE when comparing different runners. Conversely, Pinnington and colleagues [122, 123] have suggested that intra-individual increases in $\dot{V}{\text{O}}_{2}$ associated with running on sand compared with on a firm surface are partially due to increased activation of the quadriceps and hamstrings muscles involved in greater hip and knee range of motion. However, as $\dot{V}{\text{O}}_{2}$ and EMG data were collected in separate studies, causal interpretations should be made with caution. Larger intra- and inter-individual variations in lower limb muscle activity duration and timing of peak activation have been reported in novice compared with experienced runners [124], suggesting that greater running exposure may alter neuromuscular control. However, longitudinal investigations are needed to confirm this.

Conflicting results have also been reported for the role of muscular coactivation in relation to RE [46–48], whereby muscular coactivation is defined as the simultaneous activation of two muscles. Heise et al. [47] found a negative relationship between RE and the coactivation of the rectus femoris and gastrocnemius, suggesting coactivation of biarticular muscles is economical, whereas Moore et al. [48] reported a positive relationship. Furthermore, muscular coactivation of the proximal agonist–antagonist leg muscles, rectus femoris and biceps femoris, has also been shown to have a positive association with RE, meaning such coactivation is detrimental to RE [46, 48]. Coactivation of the proximal thigh antagonist–agonist muscles occurs during the loading phase of stance as the knee flexes. Without such coactivation, it is likely that the leg would collapse [125], but essentially the muscles are performing opposing movements. Using two muscles to control such a movement would therefore incur a greater metabolic cost than using one muscle, potentially decreasing the efficiency of the SSC.

Investigations into the effect of orthotics on muscular activation during ground contact and RE have provided inconsistent findings. Kelly et al. [126] reported that alterations to muscular activity when wearing orthotics during a 1-h run were not accompanied by changes in RE. Contrastingly, Burke and Papuga [127] observed improvements in RE when runners ran in custom-made orthotics rather than shoe-fitted insoles, yet there were no changes in lower limb muscular activity. However, the mass of the different orthotics used by Burke and Papuga [127], and the potential effect the orthotics had on running biomechanics, were not assessed and may have influenced their findings.

### Shoe–Surface Interaction Factors

There is a general consensus that running in traditional running trainers is detrimental to RE compared with running barefoot or in lightweight, minimalist trainers, due to the added shoe mass [49–52, 128, 129]. A recent meta-analysis suggested that a shoe mass (per pair) of less than 440 g does not affect RE, but a shoe mass greater than 440 g negatively affects RE [129]. However, when shoe mass is taken into account, evidence regarding footwear effects on RE is equivocal due to different methodologies used. Mathematically correcting for different footwear mass when expressing $\dot{V}{\text{O}}_{2}$ in relative terms supports the above statement that running in traditional trainers is detrimental to RE compared with barefoot or minimalist footwear running [50]. However, strapping weights equal to the mass of a shoe to participants’ feet results in either similar RE [52] or worse RE when barefoot compared with shod [49]. One reason for this discrepancy is that mathematically adjusting $\dot{V}{\text{O}}_{2}$ technically adjusts the whole body’s mass rather than the foot’s mass and does not take into account the decrease in lower limb moment of inertia. When the foot’s CoM is altered (weights strapped to the top of foot) $\dot{V}{\text{O}}_{2}$ is worse when barefoot [49], but when the foot’s CoM is unchanged (weights evenly distributed on the foot), $\dot{V}{\text{O}}_{2}$ is similar between barefoot and shod conditions [52]. Therefore, changes to lower limb moment of inertia, and not just shoe mass, appear to affect RE. Findings from Scholz et al. [130] support this notion by showing greater lower limb moment of inertia was associated with higher $\dot{V}{\text{O}}_{2}$. Other shoe characteristics, such as stiffness [131], comfort [132], and cushioning [133], are likely to effect RE and thus, may have also contributed to the equivocal findings regarding footwear effects on RE when shoe mass is taken into account. However, if shoe mass is not adjusted for, running barefoot or in lightweight, minimalist trainers improves RE compared with traditional running trainers (shoe mass >440 g).

Changing footwear can also change the level of cushioning underfoot. Frederick et al. [134] proposed the ‘cost of cushioning’ hypothesis, stating that actively cushioning the body whilst running may incur a metabolic cost. Therefore, shoes with limited cushioning or no cushioning (such as being barefoot) would result in an individual having to actively cushion the body using the lower limb muscles [117] and lead to an increase in $\dot{V}{\text{O}}_{2}$. Some evidence to support this claim is provided by Franz et al. [49], who found that running in shoes with increasing mass had a lower metabolic power demand than running barefoot with increasing mass strapped to their feet. These results therefore show that running without cushioning has a higher metabolic demand than running with cushioning, even when added shoe mass is similar. However, results from Divert et al. [52] suggest it may be mechanical energy that is increased rather than $\dot{V}{\text{O}}_{2}$ when barefoot. This means that barefoot running leads to mechanical efficiency improvements due to greater work being done for the same $\dot{V}{\text{O}}_{2}$ compared with shod running.

Further, it appears there is an ‘optimal’ level of surface cushioning for good RE. When running barefoot on a treadmill, 10 mm of surface cushioning was more beneficial for RE than no surface cushioning and 20 mm of surface cushioning [53]. When considering natural running terrain, Pinnington and Dawson [122] found running on grass elicited a lower $\dot{V}{\text{O}}_{2}$ than running on sand. This is likely due to the damping effects of sand, leading to an increase in mechanical work done during stance [135]. Therefore, a firmer surface that returns the energy it absorbs will benefit a runner’s RE. Moreover, a firm surface with reduced stiffness, and thus greater compliance, will return more energy due to the surface’s elastic rebound and improve RE [111].

This theory can also be applied to running shoes, as Worobets et al. [54] showed that a softer shoe, which was more compliant and lost less energy during impact than a control shoe, improved RE. Additionally, shoes with a high forefoot bending elasticity can increase propulsive force and reduce contact time and gastrocnemius muscle activation during slow (<3 m·s^−1^), but not medium (3.1–3.9 m·s^−1^), running speeds compared with a flexible forefoot region [136]. Such shoes may therefore improve RE due to enhancing propulsion; however, no $\dot{V}{\text{O}}_{2}$ data were gathered during the study, so direct associations cannot be made. Consequently, it is likely that a medium level of cushioning, that returns energy, is beneficial for RE compared with the shoe–surface cushioning being too compliant or too hard.

Footwear (or lack of) can also affect running biomechanics. Several modifications to running biomechanics may potentially benefit RE, whilst others may not. For example, in comparison with shod running, barefoot running can shorten ground contact time and stride length [49–52, 128, 137–140], increase knee flexion at initial contact [139], increase leg stiffness [52, 139, 141, 142], decrease vertical oscillation [50, 138], increase propulsive force [143], and reduce plantarflexion at toe-off [50, 139]. The most commonly cited change when running barefoot is a more anterior foot strike pattern brought about by a flatter foot, such as switching from a rearfoot to a forefoot strike pattern [50, 98, 137, 139, 140, 142, 144]. However, evidence shows many confounding variables affect foot strike, including speed [97, 145], surface stiffness [146], stride length [50], and familiarization with barefoot running [147]. Therefore, footwear (or lack of) alone cannot explain changes in foot strike. Based on the several findings above, it can be suggested that acute exposure to running barefoot may be beneficial for RE, especially if performed on a surface with a medium level of cushioning. Aside from acute exposure, the effect of individual adaptations due to short- and long-term exposure to barefoot running on RE and running biomechanics is currently unknown.

### Trunk and Upper Limb Biomechanical Factors

The relationship between RE and trunk and upper body biomechanics has received limited research attention compared with lower limb biomechanics. Swinging the arms during running plays an important role as it contributes to vertical oscillation [55, 56]; counters vertical angular momentum of the lower limbs [148]; and minimizes head, shoulder, and torso rotation [149, 150]. Eliminating arm swing by placing the hands on top of the head can be detrimental to RE [41, 149], whilst placing the hands behind the back or across the chest has provided inconsistent findings [41, 56, 63, 149, 150]. However, there is no evidence to suggest that individuals can alter arm kinematics to improve RE and thus, running performance. Therefore, based on current evidence, individuals are encouraged to maintain their natural arm swing whilst running.

Suppressing arm swing can alter several lower limb biomechanics and kinetics. For example, restraining the arms behind the back and across the chest decreases peak vertical force, increases peak hip and knee flexion angles during stance, and reduces knee adduction during stance [151]. These biomechanical changes appear to be due to the loss of arm motion rather than the body’s CoM moving position [151], suggesting that arm motion plays an integral role in an individual’s running technique. Further, the greater knee flexion and reduced peak vertical force observed when arm swing is suppressed suggests that leg stiffness decreases, which may explain the change in RE found in some studies [41, 56, 149]. However, currently, the relationship between leg stiffness and arm motion during running is unknown.

It has been suggested that a forward trunk lean during running improves RE [58], based on findings from Williams and Cavanagh [42]. Yet, a forward lean has also been implicated as detrimental to RE. Hausswirth et al. [57] compared the $\dot{V}{\text{O}}_{2}$ during a marathon run (2 h, 15 min) with that during a 45-minute run and found the marathon run had a higher $\dot{V}{\text{O}}_{2}$ and greater forward trunk lean. However, this finding should be interpreted in light of the other modifications to running biomechanics when comparing the marathon run with the 45-min run, such as the 13 % shorter stride lengths. It is possible that shortening the stride lengths by this amount incurred the highest $\dot{V}{\text{O}}_{2}$ rather than the forward lean. Additionally, the biomechanical changes could be due to muscular fatigue resulting from the difference in running time between the two conditions (1 h, 30 min), meaning muscular fatigue could have led to increases in $\dot{V}{\text{O}}_{2}$.

For women runners, breast kinematics also have the potential to affect RE and running biomechanics. Evidence shows that breast kinematics can affect running kinetics [152], trunk lean via changes in breast support [153], and lower limb biomechanics, in particular knee angle and step length [154]. These findings imply there may be alterations to RE, particularly if the changes in step length are greater than 3 % of the preferred step length. Further work that simultaneously assesses RE, breast kinematics, breast support, and lower limb biomechanics is warranted to assess whether there is a direct association between the measures.

## Simultaneously Modifying Running Biomechanics and Running Economy Through Training

Short- and mid-term training interventions (3–12 weeks) have been conducted to assess relationships between running biomechanics and RE. But to date, no long-term training interventions have been performed. Early interventions primarily focused on spatiotemporal factors, with Morgan et al. [155] showing that trained runners with uneconomical stride lengths could be retrained using audio-feedback over 3 weeks to produce mathematically derived optimal stride lengths and improved RE. In contrast, Messier and Cirillo [95] failed to find improvements in RE when using verbal and visual feedback for 5 weeks to change specific running biomechanics, such as longer stride lengths, shorter ground-contact time, and reduced vertical oscillation. However, optimal stride length was not mathematically determined prior to the intervention, meaning suitable procedures were not used and several running biomechanics either were not modified or, in the case of vertical oscillation, actually increased after the intervention. Bailey and Messier [156] also found that if runners were able to freely choose their stride length over 7 weeks, there was no change in RE. Similarly, if runners were restricted to their initial freely chosen stride length over 7 weeks, RE was unaffected [156].

Interventions concerned with instructing runners to retrain their running biomechanics towards a specific global running technique, such as Pose, Chi and midstance to midstance running, has generally resulted in either no improvement in RE [62, 85] or a worsening of RE [157]. Whilst these techniques are often advocated as efficient forms of running [157, 158], and all the interventions led to modified running biomechanics, currently there appears to be no evidence to substantiate the claims that they benefit RE. It is conceivable that the failure of global running techniques to improve RE is because they are not targeting the right running biomechanics or because they are trying to change too many at the same time.

Running gait retraining has also focused on reducing injury risk [159–162], but only one study has assessed the effect of such retraining on RE as well [163]. Clansey et al. [163] provided trained runners with gait re-training using real-time visual feedback over 3 weeks to modify impact-loading variables associated with tibial stress fracture risk. Runners reduced peak tibial acceleration and loading rates without changing RE. Thus, gait re-training to reduce injury risk can be performed without necessarily affecting running performance. This is possibly because the gait alterations were predominantly during the impact phase and have minimal effect on RE, as individuals increased plantarflexion at initial contact and exhibited a more anterior foot strike.

**Fig. 4:**
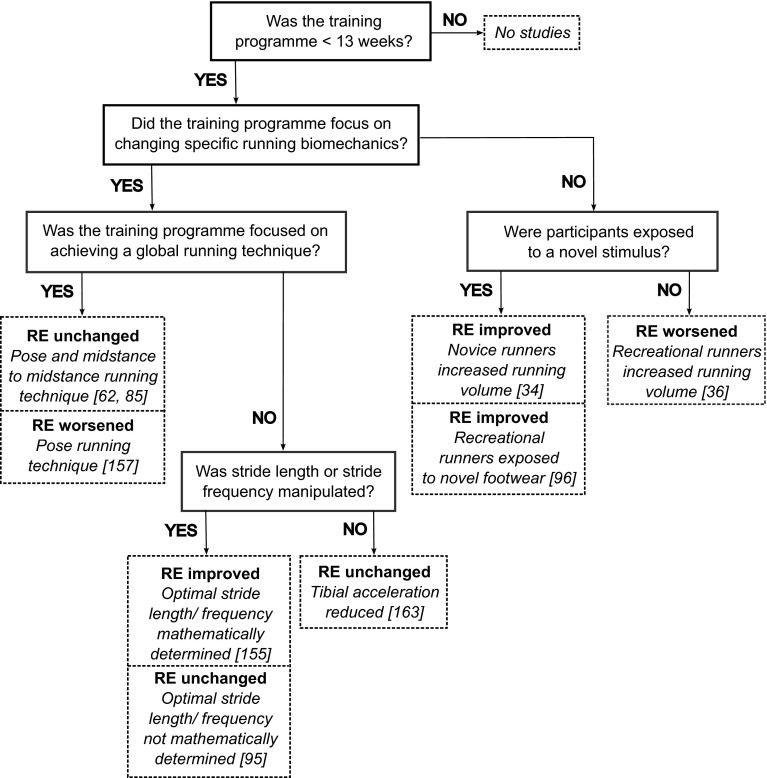
Summary of the training programs that have simultaneously measured running economy and running biomechanics. The effect on running economy is denoted in bold. *RE* running economy

Moore et al. [34] reported that novice runners could self-optimize their running gait over 10 weeks of running training, with 94 % of the variance of change in RE explained by less knee extension at toe-off, a later occurrence of peak dorsiflexion, and slower eversion velocity at initial contact. Furthermore, trained, habitually shod runners can improve their RE when running in minimalist footwear after a 4-week intervention exposing them to running in minimalist footwear [96]. Although very few running gait parameters were assessed by Warne and Warrington [96], runners did exhibit a more anterior foot strike when more economical. Whilst collectively these results support short-term biomechanical self-optimization to running training, a previous investigation failed to find RE improvements and biomechanical changes in trained runners after 6 weeks of running [36]. Consequently, novice runners may be more responsive to self-optimization in the short-term than trained runners; however providing trained runners with a novel stimulus, such as different footwear, can lead to short-term self-optimization. Thus, self-optimization is a physiological adaptation to running acquired through greater experience of the stimulus. For trained runners, the majority of this physiological adaptation may have already occurred. A summary of how training interventions have affected RE is presented in Fig. 4.

## Is there an Economical Running Technique?

Based on the literature, several modifiable factors that can potentially improve RE have been identified, as well as factors that have conflicting or limited findings regarding their relationship with RE (Table 1). From this summary, it is clear that biomechanics during ground contact play an important role. Furthermore, evidence shows that many of the running biomechanics identified occur during propulsion, suggesting that this phase has the strongest direct links with RE. However, theoretical deceleration strategies, such as short braking times and minimizing the speed lost during braking, may translate to more economical strategies in the propulsive phase and mediate the relationship between propulsion and RE. Therefore, utilizing the principles of the SSC is encouraged.

**Table 1:** Modifiable intrinsic and extrinsic running biomechanics and their effect on running economy

Evidenced effect on RE	Intrinsic				Extrinsic
	Spatiotemporal	Kinetics	Kinematics	Neuromuscular	
Beneficial	Self-selected stride length (minus 3 %)	Greater leg stiffness	Less leg extension at toe-off	Low muscle activation during propulsion	Firm, compliant shoe-surface interaction
	Low vertical oscillation	Alignment of GRF and leg axis during propulsion	Large stride angle	Low agonist–antagonist coactivation	Barefoot or lightweight shoes (<440 g)
		Low lower limb moment of inertia	Maintain arm swing		
Conflicting	Ground contact time	Impact force	Trunk lean	Biarticular coactivation	Orthotics
	Swing time	Anterior–posterior forces			
Limited or unknown	Horizontal distance between the foot and CoM at initial contact	Impulses	Swing phase	Vastus medialis preactivation	
	Braking/deceleration time		Foot-strike pattern		
	Speed lost during ground contact		Breast kinematics		

*CoM* centre of mass, *GRF* ground reaction force, *RE* running economy

Considering the empirical evidence, one economical running strategy could be aiming to shorten ground-contact times whilst maintaining stride frequency, which may facilitate greater leg stiffness, larger stride angles, and longer swing times. However, such a strategy may increase vertical oscillation and encourage greater muscular activity during propulsion. Another strategy could involve aligning the resultant GRF more closely with the leg axis during propulsion. This may help minimize muscular activity and agonist–antagonist coactivation and could be produced as a result of reducing leg extension at toe-off.

An experienced runner’s naturally chosen stride length is self-optimized to within 3 % of the mathematically derived optimal. Deviating between naturally chosen and mathematically optimal will only have a negligible effect on RE. However, novice runners have not acquired the running experience necessary to self-optimize as effectively. Therefore, a short-term running training program for novice runners can lead to running biomechanics being modified to benefit RE. However, long-term running training has seldom been investigated. Consequently, longitudinal investigations assessing the development of running biomechanics in both novice runners and experienced runners are required to better understand self-optimization for RE improvements.

Notwithstanding the identified modifiable factors affecting RE, prescribing an economical way of running has its limitations based on the current empirical evidence. The majority of studies have used cross-comparison methodologies or are restricted to one running population. Additionally, it is evident from the numerous studies analyzing intra-individual changes that group differences, which statistically hold more power, provide limited conclusions of modifications to running biomechanics [88, 119, 164]. Also, very few studies have assessed running biomechanics during the swing phase, even though current findings indicate the position of the CoM and leg during this phase may be crucial to conserving energy and reducing $\dot{V}{\text{O}}_{2}$. Exploring running biomechanics during swing and the interaction with stance-phase biomechanics is recommended in future work. Furthermore, the role of unmodifiable factors and how they may interact with modifiable factors is an area requiring investigation. For example, Cavanagh and Williams [40] reported that individuals with long legs had a larger increase in $\dot{V}{\text{O}}_{2}$ when shortening their strides compared with lengthening them. In contrast, individuals with shorter legs had a larger increase in $\dot{V}{\text{O}}_{2}$ when lengthening their stride than when shortening it.

Biomechanical case studies of economical runners have not been published, but could provide interesting findings if an in-depth runner profile was provided. Such a profile would need to encompass factors such as running biomechanics, anatomical structures, functional capacity (e.g., flexibility, muscular strength, and stiffness), shoe degradation, injury history, and training protocols [165]. Whilst only the former have been discussed here, the interaction between an individual’s anatomical structures—such as foot morphology, leg length, and tendon stiffness—and their running biomechanics is likely to be influential upon RE. This is certainly a direction for future research to pursue, as it could identify novel relationships and interactions that inform larger, cohort studies.

## Conclusion

One of the determining factors of running performance is RE. Modifiable running biomechanical factors that affect RE include spatiotemporal factors, lower limb kinematics, kinetics, neuromuscular factors, shoe–surface interactions, and trunk and upper limb biomechanics. Several intrinsic factors that appear to benefit RE are a self-selected stride length with a 3 % shorter stride length range, lower vertical oscillation, greater leg stiffness, low lower limb moment of inertia, alignment of the GRF and leg axis vectors, less leg extension at toe-off, larger stride angles, maintaining arm swing, low muscle activation during propulsion, and low antagonist–agonist thigh coactivation. In regards to extrinsic factors, better RE was found to be associated with a firm, compliant shoe-surface interaction and being barefoot or wearing lightweight shoes. Other modifiable biomechanical factors, such as ground contact time, impact force, anterior–posterior forces, trunk lean, lower limb biarticular muscle coactivation, and orthotics, presented inconsistent relationships with RE. Collectively, the evidence shows that many of the running biomechanics identified occur during propulsion, suggesting that this phase has the strongest direct links with RE. However, recurring methodological problems exist within the literature, such as cross-comparisons, assessing variables in isolation, and acute to short-term interventions. Further, intra-individual differences due to unmodifiable factors limit the findings of cross-comparisons, and future research should look to investigate longitudinal interventions and assess runners on an individual basis. Consequently, recommending an economical running technique should be approached with caution. Directions for further work within the field should focus on a synergistic approach to assessing kinetics as well as integrated approaches combining $\dot{V}{\text{O}}_{2}$, kinematics, kinetics, and neuromuscular and anatomical aspects to increase our understanding of economical running technique.
